# Cardiovascular disease market set to grow very slowly to $146.4 billion by 2022, says GBI Research

**Published:** 2016

The cardiovascular disease market, which includes hypertension, dyslipidaemia and thrombotic events, is set to grow from $129.2 billion in 2015 to $146.4 billion by 2022, at a very modest compound annual growth rate of 1.8%, according to business intelligence provider GBI Research.

The company’s latest report states that this relative stagnation can be attributed to major product approvals coinciding with key patent expirations. Within cardiovascular disease there are a number of blockbuster products that have recently gone off-patent, and others are expected to in the coming years, many of which belong to significant players.

For example, the current market leader, AstraZeneca’s Crestor (rosuvastatin), generated around $7 billion in 2011, with revenues expected to drop sharply following the expiration of its patent on 8 July 2016. Total annual revenues are forecast to be around $1.3 billion in 2022.

Thomas Jarratt, associate analyst for GBI Research, explains: ‘Unlike AstraZeneca, some key players will experience revenue growth resulting from the introduction of new products to market. In particular, Sanofi’s Praluent (alirocumab) is expected to help mitigate losses associated with falling revenues of its key products Lovenox (enoxaparin) and Plavix (clopidogrel).

‘Novartis’ heart-failure drug Entresto was introduced to market in July 2015, and GBI Research expects its revenues to increase dramatically during the forecast period. Entresto is a combination drug, which has shown efficacy in clinical trials. Coupled with a high cost, which amounts to over $4 500 annually per patient, the drug contributes to a very high revenue forecast of $5.7 billion by 2022.’

The sheer number of expirations and approvals means the structure of the market will shift significantly. Current market leader AstraZeneca is set to mitigate the damage associated with the introduction of generic Crestor through the rising revenues attributed to its antiplatelet drug Brilinta.

Jarratt continues: ‘the market shares of Sanofi and Novartis are expected to increase strongly over the forecast period, leading to Sanofi becoming market leader, and both brands achieving revenues in excess of $7 billion by 2022.’

